# 伴NUP98基因重排急性髓系白血病5例

**DOI:** 10.3760/cma.j.issn.0253-2727.2023.10.015

**Published:** 2023-10

**Authors:** 锦洁 高, 菲 董, 伟 赵, 朕豪 张, 艳芳 王, 明霞 朱, 晶 王, 红梅 景, 晓燕 克

**Affiliations:** 北京大学第三医院血液内科，北京 100191 Department of Hematology, Peking University Third Hospital, Beijing 100191, China

例1，男，63岁，因发热伴下肢关节疼痛就诊。血常规：WBC 169×10^9^/L，HGB 91 g/L，PLT 61×10^9^/L，外周血原始细胞80％；骨髓穿刺干抽，骨髓细胞形态学：粒系占92.0％，原始粒细胞占74.5％，过氧化物酶（POX）染色原始幼稚细胞阳性，酯酶染色阴性。外周血染色体核型：46，XY，t（5;15）（q31;q26.1），t（7;11）（p15;p15）[20]。荧光原位杂交（FISH）检测：NUP98基因双色分离探针重排阳性百分率为90％。NUP98-HOXA9融合基因定性阳性。二代测序（NGS）检出GATA2 p.Ala372Thr位点非同义突变（45.68％），KRAS p.Lys117Asp位点非同义突变（2.32％），NRAS p.Gly13Asp位点非同义突变（11.49％），NRAS p.Gly12Asp非同义突变（26.78％），WT1 p.Ala382f移码型插入突变（2.34％），诊断急性髓系白血病（AML）M_2_。予IA（去甲氧柔红霉素、阿糖胞苷）联合维奈克拉（100 mg第4天，200 mg第5天，300 mg第6～10天）方案诱导治疗1个疗程后，取得形态学、流式细胞学完全缓解（CR），NGS未再检出相关基因突变，FISH检测NUP98基因重排阳性率下降至6.4％。再次予原方案1个疗程后复查骨髓，仍保持形态学、流式细胞学CR，FISH未再检出NUP98基因重排。继续予去甲氧柔红霉素、中剂量阿糖胞苷联合维奈克拉巩固治疗2个疗程后保持CR状态。后续进行亲源单倍体异基因造血干细胞移植，预处理方案为：RIC-mBU3/CY-FLU+ATG，回输供者CD34^+^造血干细胞2.72×10^6^/kg，单个核细胞（MNC）5.36×10^8^/kg，+14 d粒细胞植入，+13 d血小板植入，移植后无明显并发症，复查骨髓保持CR状态，供者嵌合率100％。目前移植后9个月，无进展生存14个月余，继续随访中。

例2，女，51岁，主因乏力1个月、四肢瘀斑3 d入院，既往弥漫大B细胞淋巴瘤病史2年，治疗后已达CR。入院血常规：WBC 17.5×10^9^/L，HGB 89 g/L，PLT 8×10^9^/L，外周血原始细胞比例17％，可见Auer小体。骨髓象：有核细胞增生明显活跃，粒系占68％，原始粒细胞占21％，早幼粒细胞14.5％。POX染色原始幼稚细胞阳性，酯酶染色阴性。流式细胞学可见22.3％异常早期髓系细胞。染色体核型：46，XX；t（1;11）（q25;p15）[25]，del（7）（q22）[25]/46,XX[1]。FISH: NUP98基因重排阳性率为94％。NGS检出KRAS p.Gly13Asp位点非同义突变（1.58％），诊断AML-M_2a_。予DA（柔红霉素、阿糖胞苷）“3+7”方案化疗，化疗后第21天复查骨髓：有核细胞增生减低，原始粒细胞占20％，早幼粒细胞占10％。流式细胞术免疫表型检测见16.79％异常早期髓系细胞，染色体核型分析：46，XX；t（1;11）（q25;p15），del（7）（q22）[20]。FISH检测：NUP98基因重排阳性率70％，考虑疾病未缓解。予CAG（小剂量阿糖胞苷、阿克拉霉素、G-CSF）联合维奈克拉［25 mg第4～5天，50 mg第6～10天（联合强效CYP3A4抑制剂预防真菌感染）］再诱导后复查骨髓：增生活跃，原始粒细胞0.5％。流式细胞术微小残留病（MRD）阴性，FISH检测NUP98基因重排阳性率下降至5％，达到CR。继续予中剂量阿糖胞苷联合维奈克拉巩固2个疗程后复查骨髓形态学、免疫学保持CR，FISH检测未再发现NUP98基因重排，NGS未检出相关基因突变。继续予柔红霉素+中剂量阿糖胞苷及维奈克拉巩固化疗，骨髓象为持续CR状态，无进展生存10个月余，继续随访中。

例3，男，45岁，因头晕、头痛伴乏力3周入院。血常规：WBC 159.42×10^9^/L，HGB 41 g/L，PLT 23×10^9^/L；骨髓象：有核细胞增生明显活跃，粒系占92％，原始粒细胞占40％，早幼粒细胞25％，偶见Auer小体，POX染色阳性。流式细胞术检测可见34.41％异常早期髓系细胞，染色体核型：46, XY, t（7;11）（p15;p15）[19]/46,XY[1]。FISH：NUP98基因重排阳性率为85％。NUP98-HOXA9融合基因定性阳性。NGS：RUNX1 p.T354fs位点缺失突变（14.96％）、GATA2 p.A372T位点突变（33.61％）、FLT3-ITD位点插入突变。诊断AML-M_2a_。予以DA（柔红霉素、阿糖胞苷）方案1个疗程后取得CR，后分别行DA、MA（米托蒽醌、阿糖胞苷）方案各1个疗程，7个月后复发，尝试CAG（阿糖胞苷、阿克拉霉素、G-CSF）、MA+地西他滨、小剂量AA（阿克拉霉素、阿糖胞苷）、HA（高三尖杉酯碱、阿糖胞苷）、HAD（HA+柔红霉素）等方案均未缓解，首次复发14个月后死亡。

例4，男，72岁，主因发热伴心悸、头晕、头痛2周入院。血常规：WBC 105.18×10^9^/L，HGB 48 g/L，PLT 18×10^9^/L；骨髓象：有核细胞增生极度活跃，粒系85.5％，原始粒细胞62.5％，早幼粒细胞占10％，POX染色原始幼稚细胞阳性；流式细胞术免疫表型：75.89％为异常早期髓系细胞。染色体核型：47,XY,t（7;11）（p15;p15）, +8[18]/46,XY[2]。FISH：NUP98基因重排阳性百分率为80％。NUP98-HOXA9融合基因定性阳性。未进行NGS检测。诊断为AML-M_2a_。予以小剂量AA方案（阿克拉霉素10 mg第1～13天，阿糖胞苷 50 mg 第1～13天）化疗1个疗程，复查骨髓评价疗效为部分缓解后离院，1个月后死亡。

例5，女，45岁，主因头晕、心悸8个月余入院。既往诊断骨髓增生异常综合征伴原始细胞增多（MDS-EB）-Ⅱ3个月，未治疗。入院血常规：WBC 3.83×10^9^/L，RBC 2.2×10^12^/L，HGB 68 g/L，PLT 22×10^9^/L；骨髓象：有核细胞增生减低，粒系占60.0％，原始粒细胞占30.0％，POX染色原始幼稚细胞阳性；流式细胞术免疫表型：26.91％为异常早期髓系细胞，染色体核型：46,XX,t（7;11）（p15;p15）[20]。FISH：NUP98重排阳性百分率86％。NUP98-HOXA9融合基因定性阳性。NGS检出NRAS p.G13Rrs121434595非同义突变（11.23％）、WT1 p.V379fs移码型插入突变（21.49％）。诊断为AML-M_2a_。予以地西他滨+CAG方案，化疗第10天因出现粒细胞缺乏、发热（体温39 °C）停止化疗，后出现意识障碍，经气管插管有创呼吸机抢救，后家属放弃治疗，于离院5 d后死亡。

讨论：在第五版WHO造血与淋巴组织肿瘤分类中，“急性髓系白血病伴NUP98重排”被归入“伴重现性遗传学异常的急性髓系白血病”类目下，成为新的独立亚型。伴NUP98基因重排的AML儿童发病率略高，约5％，在成人AML中发病率低，为1％～2％。这类患者对诱导化疗反应率低，其总生存期、无白血病生存期明显低于NUP98基因重排阴性的AML患者。目前尚无针对NUP98基因的靶向药物或统一的标准治疗方案，此类患者从单纯传统化疗中的获益程度有限。我院5例患者基因重排类型主要为NUP98-HOXA9，FAB分型均为M_2_型，与国内外研究报道相一致。例1、例2首次尝试在标准化疗基础上联合维奈克拉作为一线诱导方案，均取得诱导1个疗程CR的良好疗效。尤其例2在前期DA方案诱导未缓解、骨髓增生低下的基础上，改用小剂量CAG方案联合维奈克拉再诱导达到CR，提示维奈克拉对提高诱导化疗反应率的重要性。

